# Respiration-Averaged CT for Attenuation Correction of PET Images – Impact on PET Texture Features in Non-Small Cell Lung Cancer Patients

**DOI:** 10.1371/journal.pone.0150509

**Published:** 2016-03-01

**Authors:** Nai-Ming Cheng, Yu-Hua Dean Fang, Din-Li Tsan, Ching-Han Hsu, Tzu-Chen Yen

**Affiliations:** 1 Departments of Nuclear Medicine, Chang Gung Memorial Hospita, Linkou, Chang Gung University College of Medicine, Taoyuan City 33305, Taiwan; 2 Department of Biomedical Engineering and Environmental Sciences, National Tsing Hua University, Hsinchu City, 30071, Taiwan; 3 Department of Biomedical Engineering, National Cheng Kung University, Tainan City, 70101, Taiwan; 4 Department of Radiation Oncology, Chang Gung Memorial Hospital, Chang Gung University College of Medicine, Taoyuan City 33305, Taiwan; National Cancer Centre Singapore, SINGAPORE

## Abstract

**Purpose:**

We compared attenuation correction of PET images with helical CT (PET/HCT) and respiration-averaged CT (PET/ACT) in patients with non-small-cell lung cancer (NSCLC) with the goal of investigating the impact of respiration-averaged CT on ^18^F FDG PET texture parameters.

**Materials and Methods:**

A total of 56 patients were enrolled. Tumors were segmented on pretreatment PET images using the adaptive threshold. Twelve different texture parameters were computed: standard uptake value (SUV) entropy, uniformity, entropy, dissimilarity, homogeneity, coarseness, busyness, contrast, complexity, grey-level nonuniformity, zone-size nonuniformity, and high grey-level large zone emphasis. Comparisons of PET/HCT and PET/ACT were performed using Wilcoxon signed-rank tests, intraclass correlation coefficients, and Bland-Altman analysis. Receiver operating characteristic (ROC) curves as well as univariate and multivariate Cox regression analyses were used to identify the parameters significantly associated with disease-specific survival (DSS). A fixed threshold at 45% of the maximum SUV (T45) was used for validation.

**Results:**

SUV maximum and total lesion glycolysis (TLG) were significantly higher in PET/ACT. However, texture parameters obtained with PET/ACT and PET/HCT showed a high degree of agreement. The lowest levels of variation between the two modalities were observed for SUV entropy (9.7%) and entropy (9.8%). SUV entropy, entropy, and coarseness from both PET/ACT and PET/HCT were significantly associated with DSS. Validation analyses using T45 confirmed the usefulness of SUV entropy and entropy in both PET/HCT and PET/ACT for the prediction of DSS, but only coarseness from PET/ACT achieved the statistical significance threshold.

**Conclusions:**

Our results indicate that 1) texture parameters from PET/ACT are clinically useful in the prediction of survival in NSCLC patients and 2) SUV entropy and entropy are robust to attenuation correction methods.

## Introduction

Despite significant advances in targeted therapy [1], the prognosis of patients with non-small cell lung cancer (NSCLC) remains dismal. ^18^F-FDG PET imaging plays an essential role in the diagnosis and staging of NSCLC. Recent years have witnessed an increased use of FDG PET imaging for clinical decision making [2]. Accordingly, maximum standardized uptake values (SUV_max_) [3, 4] and total lesion glycolysis (TLG) [5, 6] have been shown to be clinically useful for predicting treatment response and clinical outcomes of NSCLC patients. Tumor heterogeneity has been generally related to poor prognosis and treatment resistance [7]. Growing evidence indicates that FDG PET texture features reflecting tumor heterogeneity may predict therapeutic response and survival in NSCLC [8–11] and numerous other malignancies [12–20]. The mining of a large number of quantitative image features has been referred to “radiomics” and holds promise as a method for identifying specific prognostic signatures [10, 21–26]. In this scenario, accurate and reproducible measurements of texture features are essential for clinical use. Unfortunately, respiratory motion during PET/CT results in degradation of image quality and hampers the correct quantification of imaging parameters [27–29]. Such deterioration is caused by the discrepancy of chest position between helical CT (HCT) and PET images [30, 31]. Rather than a snapshot of the respiration cycle (as in HCT images), PET scans are the results of the average of respiratory cycles. Notably, the temporal difference between PET and CT ultimately introduces misalignment artifacts in PET images. To address this issue, attenuation correction of PET images with respiration averaged CT (ACT) has been utilized. Rather than providing a motionless CT image, ACT simulates the PET acquisition process by averaging the signal during a respiratory cycle from multiple low-dose cine CT images. Previous studies have shown that ACT correction can improve the quality of PET images by reducing misalignments and optimizing the quantification of SUV [31–34]. Nevertheless, the utility of ACT for correcting PET texture features has not been thoroughly investigated. In addition, data on the potential prognostic impact of different PET texture features in NSCLC patients remain scarce. Because of the increasing use of ^18^F-FDG PET/CT in clinical trials, an analysis of the variability related to attenuation correction is worthy of investigation. We therefore designed the current study to compare attenuation correction of PET images with HCT and ACT in NSCLC with the goal of investigating the impact of respiration-averaged CT on FDG PET texture parameters.

## Materials and Methods

### Patients

The Institutional Review Board of the Chang Gung Memorial Hospital at Linkou approved the study protocol (102-3810B). Written informed consent was obtained from all patients before ACT. The study population consisted of patients with pathologically-proven NSCLC who were scheduled to undergo definitive treatment with curative intent. All of the study participants underwent ^18^F-FDG PET for disease staging. The treatment approach was as follows: 1) radical surgery for stage IA patients, 2) radical surgery plus adjuvant chemotherapy for stage IB and IIA patients, 3) neoadjuvant therapy (chemotherapy, radiotherapy, or concurrent chemoradiotherpy [CCRT]) and operation for stage IIB and IIIA patients, and 4) chemotherapy, radiotherapy, or CCRT and operation (in presence of resectable disease) for stage IIIB patients. Patients with M1 disease were excluded. Patients were staged according to the 2010 (7^th^ edition) American Joint Committee on Cancer (AJCC) staging system. We retrospectively reviewed the clinical charts to extract the general characteristics and the clinical outcomes of the participants. Disease-specific survival (DSS) − defined as the time from diagnosis to NSCLC-related death − served as the main outcome measure.

### PET/CT imaging protocol

Patients were asked to fast 6 h before examination. According to our institutional policy, patients with blood glucose levels greater than 200 mg/dL had their scan rescheduled. All participants were imaged using the same PET/CT scanner (Discovery ST16, GE Healthcare). Scans were obtained 50 min after intravenous FDG administration. The injected dose of FDG was calculated according to body weight and ranged between 370 and 555 MBq. HCT data were acquired with the following settings: 120 kV, automatic mA (range: 10−300 mA), pitch 1.75:1, collimation 16×3.75 mm, and rotation cycle 0.5 s. Whole-body PET emission scans were performed in the 2D mode and acquired from the skull to the mid-thigh. Following HCT and PET acquisition, a low-dose cine CT was performed using the following settings: 120 kV, automatic mA (range: 10−25 mA according to the patient’s body weight), rotation cycle 0.5 s, collimation 8×2.5 mm, and cine duration 5.9 s. The goal was to include lung fields bilaterally from the pulmonary apex to the dome of the liver [32, 35–37]. Ten phases of cine CT images were averaged to obtain ACT. No intravenous contrast enhancement was used, and imaging was performed in the free-breathing state. No pre- or in-scan breathing coach for respiratory control was used. Attenuation correction of PET images was performed with both HCT and ACT using the same PET data [30, 32, 35]. PET emission data were reconstructed with attenuation correction using both HCT and ACT attenuation maps. Transaxial emission images were reconstructed using ordered subsets expectation maximization (OSEM) with 4 iterations and 10 subsets. PET images were reconstructed on a 128 × 128 image matrix with a voxel size of 4.46 × 5.46 × 3.27 mm^3^ for both PET/HCT and PET/ACT. An additional dose of 2.5 mSv (5 mGy) was used for patients with a body weight > 70 kg [38].

### PET/CT image data analysis

The PMOD 3.3 software package (PMOD Technologies Ltd, Zurich, Switzerland) was used for tumor segmentation. We applied two methods for tumor segmentation, i.e. (1) the adaptive threshold approach in the exploratory analysis and (2) 45% of SUV_max_ (T45) for validation purposes. The adaptive threshold was determined by using a mean intensity of voxel contoured by 70% of the tumor SUVmax *(I*_*mean 70%*_*)* plus the background mean SUV [39]. The aortic arch was used for background measurement and none of the study participants had aortic arch invasion. Two authors (N.M.C. and T.C.Y.) contoured the aortic arch using 1 cm^3^ cubic volumes of interest (VOI) and results were averaged. Care was taken to exclude calcified regions in the aortic arch. Finally, the adaptive threshold was calculated according to the following formula: 0.15 × (*I*_*mean 70%*_) + background. The T45 approach has been previously utilized for delineation of NSCLC tumors [8]. SUV_max_, mean SUV, and texture features were determined using the tumor VOI. TLG was calculated as follows: TLG = mean SUV × metabolic tumor volume (MTV) [40]. We did not analyze nodal lesions because of their small size.

Histogram analysis, normalized grey-level co-occurrence matrix (NGLCM) [41, 42], neighborhood grey-tone difference matrix (NGTDM)[43], and grey level size zone matrix (GLSZM) [44] were applied for calculation of PET texture features. Because numerous texture features have been reported [12, 45, 46], we specifically focused on those utilized for predicting survival in patients with malignancies. Several texture parameters, including entropy, uniformity, homogeneity and dissimilarity from NGLCM [11, 14, 47, 48], grey-level nonuniformity (GLNU), zone-size nonuniformity (ZSNU), high grey-level large zone emphasis (HGLZE) from GLSZM [14, 19], and NGTDM based coarseness, busyness, contrast and complexity [8] have been used for survival prediction in patients with NSCLC, esophageal cancer, and head and neck malignancies. In addition, we evaluated SUV entropy based on histogram analysis because of its robustness due to different reconstruction settings [45, 49]. A total of 12 different texture features were examined. The intensity values of the recorded VOIs were initially resampled into 64 bins to normalize images and reduce noise for the calculation of texture features [13]. The computations for texture features were performed using the Chang-Gung Image Texture Analysis toolbox (CGITA) implemented under MATLAB 2012a (Mathworks Inc., Natick, MA, USA). The details on mathematical models for texture matrices and the calculation process have been previously described in detail [14, 50].

### Statistical analysis

Because most texture features showed a skewed distribution, the non-parametric Wilcoxon signed-rank test was used for paired comparisons of PET/HCT and PET/ACT parameters. The reciprocal associations of texture features in PET/HCT and PET/ACT were examined using intraclass correlation coefficients (ICC). Precision was defined by half of the width of the 95% confidence intervals (CIs) and used as an indicator of reliability. Bland-Altman analysis was used for comparing two measurements. The differences between the two parameters (i.e., PET/ACT values minus PET/HCT values) were plotted against their average (e.g. mean of PET/HCT and PET/ACT values) and reported as percentage. The lower and upper reproducibility limits (LRL and URL, respectively) were calculated as ± 1.96 standard deviations (SD). Variations were defined as the range between LRL and URL. In an exploratory analysis, the evaluation of texture parameters was based on a step-forward process. The median follow-up time in the entire study cohort was 26.2 months (range: 2.5−74.8 months), whereas it was 59.0 months (range: 40.4−74.8 months) in patients who survived. Because all of the enrolled cases were followed up of at least 3 years or until death, receiver operating characteristic (ROC) curves were initially used to identify the image features associated with 3-year DSS. All of the parameters with an area under curve different from 0.5 were selected for further analyses. The optimal cutoff values were identified by determining the point where the sum of sensitivity and specificity (Youden’s index) was maximum. Dichotomizing patients according to the optimal cutoff values were used in subsequent univariate and multivariate Cox regression analyses. Because of the high collinearity among different texture features, we constructed multivariate Cox regression models to include only one texture parameter and the following covariates: age, cell type (adenocarcinoma *vs*. non-adenocarcinoma), AJCC stage (stage I, II *vs*. stage III), and radical surgery (yes *vs*. no). All calculations were performed with the PASW Statistics 18 software package (SPSS Inc., Chicago, IL, USA). After application of the Bonferroni correction, a *P* value < 0.017 (i.e., 0.05/3) was considered statistically significant.

## Results

### Patients

Between July, 2007 and June, 2009, a total of 56 consecutive patients (36 males, 20 females; median age: 68 years; age range: 34−84 years) were enrolled. The median follow-up time in the entire study cohort was 26.2 months (range: 2.5−74.8 months), whereas it was 59.0 months (range: 40.4−74.8 months) in patients who survived. The clinical characteristics of the study participants are shown in Table 1. The most common histological type was adenocarcinoma (n = 31, 55.4%), and the majority of patients were diagnosed at advanced stages (stage IIIA or IIIB, n = 36, 64.2%). Twenty-two (39.3%) tumors were located in the lower pulmonary lobes, whereas 34 (60.7%) were located in the upper or right middle lobes. A total of 29 patients (51.8%) received radical surgery. The median glucose level before FDG PET imaging was 93 mg/dL (range: 65−151 mg/dL). There were no significant interobserver differences in background activity for both PET/HCT (observer 1 *vs*. 2: 1.60 ± 0.30 vs. 1.62 ± 0.31, P = 0.325) and PET/ACT (1.65 ± 0.34 *vs*. 1.65 ± 0.32, P = 0.960). No differences were noted in the values of resulting adaptive thresholds (2.88 ± 0.80 *vs*. 2.91 ± 0.81, P = 0.318 and 2.96 ± 0.82 *vs*. 2.96 ± 0.83, P = 0.954 for PET/HCT and PET/ACT, respectively). Using the mean threshold for tumor delineation, the median MTV for PET/HCT and PET/ACT were 26.19 cm^3^ and 26.58 cm^3^, respectively (Wilcoxon signed-ranks test, *P* = 0.426).

**Table 1:** Clinicopathological characteristics of the study patients.

Characteristic	n (%)
**Sex**	
** Male**	36 (64.3)
** Female**	20 (35.7)
**Age (years)**	
** Range**	34−84
** Median**	68
**Sites of tumors**	
** Lower lobe**	22 (39.3)
** Other lobes**	34 (60.7)
**TNM stage**	
** T1**	5 (8.9)
** T2**	28 (50.0)
** T3**	3 (5.4)
** T4**	20 (35.7)
** N0**	20 (35.7)
** N1**	10 (17.9)
** N2**	19 (33.9)
** N3**	7 (12.5)
**AJCC Stage**	
** IA**	3 (5.4)
** IB**	11 (19.6)
** IIA**	2 (3.6)
** IIB**	4 (7.1)
** IIIA**	11 (19.6)
** IIIB**	25 (44.6)

### PET image analysis

**Fig 1:**
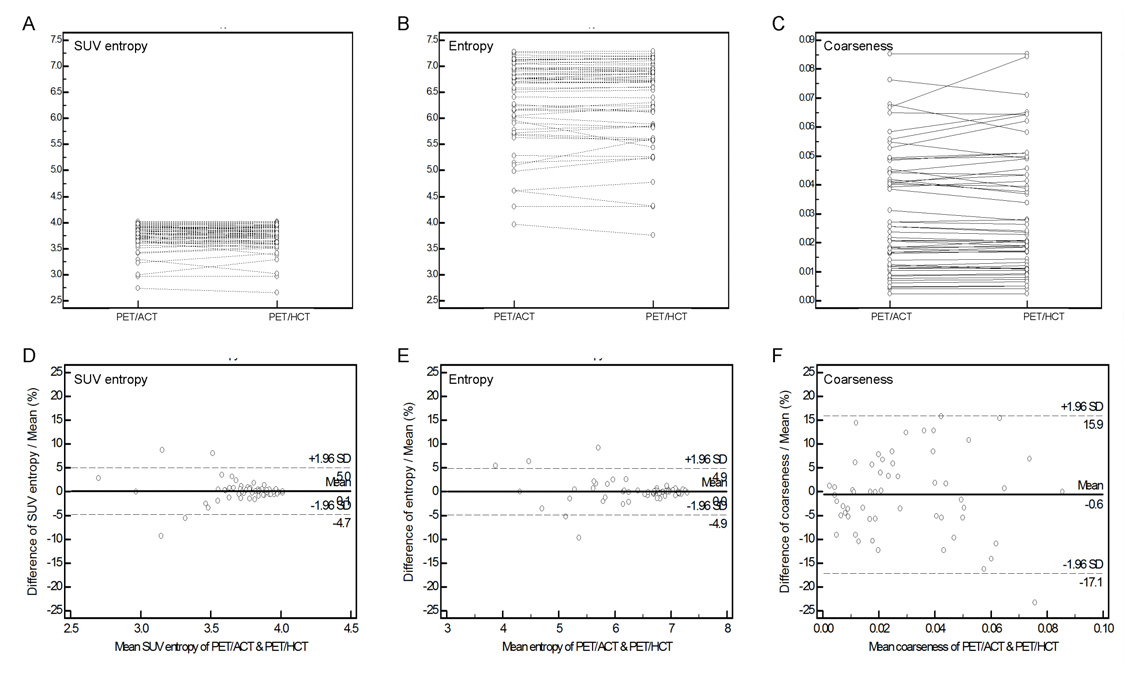
Dot-and-line diagrams of SUV entropy (A), entropy (B), and coarseness (C). Results of Bland-Altman analysis of SUV entropy (D), entropy (E), and coarseness (F).

The results of Wilcoxon sign-ranks tests revealed that PET/ACT yielded significant higher SUV_max_, SUV mean, and TLG values. However, all of the texture parameters did not show significant differences (Table 2). Specifically, SUV_max_ of tumors located in the lower lung were significantly higher in PET/ACT, but similar values were noted for tumors arising in other sites (S1 Table). Despite differences in SUV_max_, SUV mean, and TLG between PET/HCT and PET/ACT, ICC analysis revealed a high degree of correlation and good precision (ICC: 0.993, 0.994, and 0.993; precision: 0.35, 0.35, and 0.40% for SUV_max_, SUV mean, and TLG, respectively). HGLZE showed the lowest levels of correlation and precision (0.919 and 4.35%, respectively). High correlation coefficients (ICC > 0.95) were generally noted for other texture features (Table 3). The variations of SUV_max_ and SUV mean in Bland-Altman analysis were 25.4% and 18.1%, respectively. The lowest level of variation was evident for SUV entropy (9.7%) followed by entropy (9.8%), as revealed in Fig 1. Among NGTDM and GLSZM parameters, coarseness and GLNU had the lowest degree of variation (33.0% and 45.2%, respectively). The highest levels of variation were noted for contrast (104.9%) and HGLZM (80.6%), S1 Fig. Variation values greater than 50% were evident for uniformity (56.6%), busyness (52.9%), complexity (67.1%), ZSNU (74.4%), and HGLZE (80.6%). High degrees of correlation between texture parameters (│ρ│ = 0.614–0.993 for PET/HCT and │ρ│ = 0.567–0.993 for PET/ACT, all *P* < 0.001) were evident for both PET/ACT and PET/HCT.

**Table 2:** Results of Wilcoxon signed-ranks tests for PET/HCT and PET/ACT parameters.

Variables	PET/HCT			PET/ACT			*P*
	Mean	SD	Range	Mean	SD	Range	
**SUV**_**max**_	11.12	5.95	3.03–36.05	11.29	6.03	3.08–36.74	0.009
**SUV mean**	5.05	2.16	2.01–13.03	5.16	2.22	2.01–13.80	<0.001
**TLG**	273.5	378.5	2.7–1802.8	287.0	403.3	2.6–1846.7	<0.001
**Texture parameters**							
**SUV entropy**	3.71	0.26	2.66–4.02	3.72	0.26	2.74–4.01	0.516
**Uniformity**	0.003	0.004	0.001–0.02	0.003	0.003	0.001–0.02	0.853
**Entropy**	6.35	0.82	3.76–7.30	6.35	0.81	3.97–7.28	0.734
**Dissimilarity**	8.83	2.71	4.85–19.13	8.84	2.76	4.65–19.21	0.766
**Homogeneity**	0.19	0.04	0.12–0.27	0.19	0.04	0.12–0.28	0.969
**Coarseness**	0.030	0.022	0.002–0.09	0.030	0.02	0.002–0.09	0.780
**Busyness**	0.222	0.296	0.021–1.528	0.224	0.303	0.020–1.503	0.411
**Contrast**	0.081	0.335	0.0004–2.336	0.075	0.307	0.0004–2.136	0.382
**Complexity**	52.35	68.94	0.98–277.8	51.26	64.22	0.95–273.4	0.256
**Grey-level nonuniformity**	9.67	11.78	1.00–67.36	9.73	11.89	1.12–66.15	0.714
**Zone-size nonuniformity**	214.9	231.6	10.2–1231.3	214.1	232.8	15.1–1262.4	0.914
**High grey-level large zone emphasis**	2564	1461	1012–7223	2685	1652	891–10488	0.154

SUV, standardized uptake value; TLG: total lesion glycolysis.

**Table 3:** Intraclass correlation coefficients and Bland-Altman analyses of PET parameters.

Variables	Intraclass Correlation Coefficient (ICC)			Bland-Altman analysis		
	ICC	95% CI	Precision (%)	Mean	Variation (%)	LRL^a^–URL^b^ (%)
**SUV**_**max**_	0.993	0.989–0.996	0.35	1.5	25.4	-11.2–14.2
**SUV mean**	0.994	0.990–0.997	0.35	2.3	18.1	-6.7–11.4
**TLG**	0.993	0.988–0.996	0.40	3.5	43.8	-18.4–25.4
**Texture parameters**						
**SUV entropy**	0.949	0.915–0.970	2.75	0.1	9.7	-4.7–5.0
**Uniformity**	0.956	0.926–0.974	2.40	0.2	56.6	-28.1–28.5
**Entropy**	0.987	0.978–0.992	0.70	0	9.8	-4.90–4.90
**Dissimilarity**	0.975	0.964–0.987	1.15	-0.1	22.7	-11.4–11.3
**Homogeneity**	0.977	0.962–0.987	1.25	-0.4	16.7	-8.7–8.0
**Coarseness**	0.982	0.970–0.990	1.00	-0.6	33.0	-17.1–15.9
**Busyness**	0.996	0.993–0.998	0.25	-0.6	52.9	-27.1–25.8
**Contrast**	0.983	0.971–0.990	0.95	-2.3	104.9	-54.8–50.1
**Complexity**	0.975	0.958–0.986	1.40	-1.0	67.1	-34.5–32.6
**Grey-level nonuniformity**	0.998	0.997–0.999	0.10	-1.5	45.2	-24.1–21.1
**Zone-size nonuniformity**	0.997	0.994–0.998	0.20	-1.2	74.4	-38.4–36.0
**High grey-level large zone emphasis**	0.919	0.865–0.952	4.35	4.5	80.6	-35.4–44.8

^a^LRL: lower reproducibility limit

^b^URL: upper reproducibility limit.

### Survival analysis

**Fig 2:**
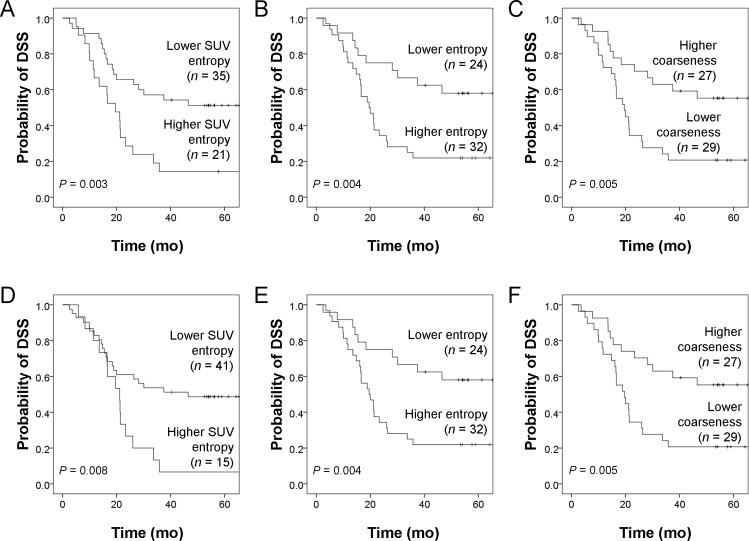
Kaplan-Meier estimates of disease-specific survival (DSS) from PET/HCT (A-C) and PET/ACT (D-F) parameters. Kaplan-Meier estimates of DSS stratified according to distinct cut-off values of PET/HCT and PET/ACT parameters. Cut-off values are shown in S2 Table. Log-rank test *P* values are also reported.

At the end of study, 36 patients died (35 of NSCLC and one of acute myocardial infarction). A total of 47 patients (83.9%) had disease progression during follow-up. Three methods (ROC curve analyses, univariate Cox regression analysis, multivariate Cox regression analysis) were used to investigate the prognostic role of texture parameters. The results of ROC curve analyses (S2 Table) revealed that SUV entropy, uniformity, entropy, coarseness, contrast, GLNU, and ZSNU from PET/HCT and PET/ACT were statistically significant. When patients were dichotomized according to the optimal cutoff values, we found a high consistency of texture parameters derived from PET/HCT and PET/ACT. Identifcal stratification results were obtained with regard to entropy and coarseness; 55 of 56 cases (98.2%) were based on the cut-off for GLNU; 54 (96.4%) for ZSNU; 53 (94.6%) for uniformity and 50 (89.3%) for SUV entropy. ICC, precision, and variation did not show significant associations with stratification consistency (Spearman’s ρ = -0.211, 0.266 and -0.248, *P* = 0.559, 0.457 and 0.490, respectively). Subsequently analyses using univariate and multivariate Cox models confirmed the significant role of SUV entropy, entropy, and coarseness from both PET/HCT and PET/ACT in the prediction of DSS (Table 4). The complete results of univariate and multivariate Cox regression analyses are shown in S5 Table. Kaplan-Meier estimates of DSS for PET/HCT and PET/ACT parameters are shown in Fig 2.

**Table 4:** Univariate and multivariate Cox regression analyses of disease-specific survival using PET parameters.

Variables	Univariate analysis		Multivariate analysis^a^	
	HR (95% CI)	*P*	HR (95% CI)	*P*
**PET/HCT**				
**SUV entropy**	2.69 (1.37–5.29)	0.004	2.69 (1.32–5.45)	0.006
**Entropy**	2.81 (1.34–5.92)	0.006	2.69 (1.23–5.89)	0.013
**Coarseness**	0.38 (0.19–0.76)	0.007	0.34 (0.15–0.79)	0.012
**PET/ACT**				
**SUV entropy**	2.49 (1.24–4.99)	0.010	2.77 (1.32–5.83)	0.007
**Entropy**	2.81 (1.34–5.92)	0.006	2.69 (1.23–5.89)	0.013
**Coarseness**	0.38 (0.19–0.76)	0.007	0.34 (0.15–0.79)	0.012

HR: hazard ratio; CI: confidence interval.

^a^Adjusted for age, cell type, radical surgery, and AJCC stage (see text).

### Validation data

**Fig 3:**
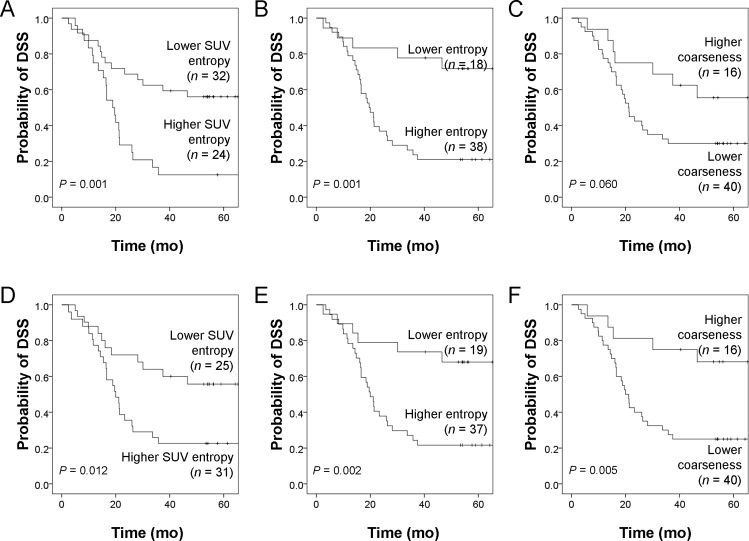
Kaplan-Meier estimates of disease-specific survival (DSS) for PET/HCT (A-C) and PET/ACT (D-F) parameters segmented by T45. Kaplan-Meier estimates of DSS rates stratified according to distinct cut-off values of PET/HCT and PET/ACT parameters. Cut-off values are shown in S2 Table. Log-rank test *P* values are also reported.

MTV and TLG delineated using the T45 method were significantly lower than those obtained using the adaptive threshold approach (Wilcoxon signed-ranks test, both *P* <0.001). Significant higher SUV_max_, SUV mean and TLG were also evident (S3 Table), whereas all of the texture parameters showed no statistically significant differences. High ICCs were identified. SUV entropy and entropy revealed the lowest degrees of variation between PET/ACT and PET/HCT, whereas contrast and HGLZE continued to show large variations (S4 Table). Using the T45 method for validation purposes, the predictive role of SUV entropy and entropy from both PET/HCT and PET/ACT for DSS was confirmed using all of the three methodologies (ROC curves as well as univariate and multivariate Cox regression analyses). However, the role of coarseness remained significant only from PET/ACT (Table 5). Kaplan-Meier estimates of DSS for these analyses are reported in Fig 3.

**Table 5:** Univariate and multivariate Cox regression analyses of disease-specific survival using PET parameters with tumor segmented with the T45 approach.

Variables	Univariate analysis		Multivariate ^a^	
	HR (95% CI)	*P*	HR (95% CI)	*P*
PET/HCT				
SUV entropy	3.16 (1.58–6.35)	0.001	3.26 (1.52–6.99)	0.002
Entropy	4.52 (1.74–11.78)	0.002	3.87 (1.47–10.22)	0.006
Coarseness	0.46 (0.20–1.05)	0.066	0.42 (0.18–1.02)	0.056
PET/ACT				
SUV entropy	2.47 (1.20–5.09)	0.014	2.81 (1.29–6.09)	0.009
Entropy	3.74 (1.54–9.09)	0.004	3.13 (1.27–7.73)	0.013
Coarseness	0.28 (0.11–0.72)	0.008	0.26 (0.10–0.69)	0.007

HR: hazard ratio; CI: confidence interval.

^a^Adjusted for age, cell type, radical surgery and AJCC stage (see text).

## Discussion

The quantification of PET images relies on accurate attenuation correction maps. However, respiration motion continues to remain a major challenge for PET/CT imaging. PET/HCT misregistration occurs when HCT imaging is performed during the inspiration [27–29, 51]. Accordingly, a displaced diaphragm by air-filled lung tissues results in an underestimation of the attenuation coefficient. Because PET/ACT can effectively reduce this issue, higher SUV_max_ values were identified in PET/ACT (especially for lesions located in the lower where a more marked respiratory motion was expected). In contrast, SUV_max_ values did not differ significant at sites different from the lower and background. Notably, texture indices obtained from PET/ACT and PET/HCT were largely similar even in the lower lung fields. Texture features are calculated using whole-tumor sampling and are useful for assessing the relationships between multiple voxels and their neighborhood (rather than a single voxel). Therefore, they are generally consistent even when different attenuation correction methods are used.

PET entropy has been shown to predict survival in patients with early-stage NSCLC [48]. In the current study, we were not only able to replicate this finding but we also showed that heterogeneous PET images were associated with unfavorable DSS. Heterogeneous images were characterized by larger values of SUV entropy and entropy. Notably, SUV entropy and entropy based on NGLCM from both PET/HCT and PET/ACT were significant predictors of survival. Moreover, SUV entropy and entropy remained consistent regardless of different PET reconstruction parameters (iteration number, FWHM, and pixel size) [49] and attenuation correction methods. NGTDM was originally developed to quantify human visual perception. A coarse image reflects the presence of a uniform intensity distribution, e.g. a homogeneous image. Although a previous study demonstrated a prognostic role of coarseness [8], this parameter shows a high extent of variation according to different segmentation methods, PET acquisition modes, and image reconstruction settings [45, 49, 52, 53]. As expected, coarseness values in this study were characterized by a marked extent of variation according to the attenuation correction method (33.0% and 55.0% for the adaptive threshold and T45 methods, respectively). Notably, coarseness was significantly associated with DSS using the adaptive threshold method. However, only a marginal association was observed when the T45 delineation without motion compensation was applied. In contrast, SUV entropy and entropy (which were characterized by a lower extent of variation) were significant in both PET/ACT and PET/HCT.

Different from our results, Yip et al. [54] reported significant differences between PET/HCT and 4D PET/CT for coarseness and busyness values. In 4D-PET/CT, 4D-CT images of five different respiratory phases are selected to match those of the corresponding 4D-PET acquired following PET/HCT. Consequently, the count of 4D-PET photons is different from that of PET/HCT, with a higher noise being evident for 4D PET (which may hamper the precise calculation of the texture features). The question as to whether such differences may have an impact on survival prediction remains open. Differently from 4D-PET/CT, PET/ACT uses the same PET images and all of the phases of PET signals are utilized. This observation may explain the limited differences in terms of texture parameters between PET/HCT and PET/ACT.

Our findings emphasize the importance of using a standardized approach for PET texture analysis [55] in NSCLC patients. It may be argued that the diversity of PET texture parameters (resulting from differences in target segmentation, rebin process, reconstruction settings, and/or terminology) may hamper the application of texture-based analysis in clinical practice [53]. However, technical advances and the implementation of randomized clinical trials will hopefully be helpful in overcoming such challenges [2, 56]. It is also possible that texture parameters (especially SUV entropy and entropy) can be useful in guiding radiotherapy. In this regard, it would be clinically relevant to assess the value of dose painting using unfixed radiation dose distribution to the tumor (based on image-guided stratification of high-risk target volumes). Although dose painting based on PET images with high SUV does not seem to be clinically useful for predicting outcomes [57], the identification of high-risk subvolumes based on PET texture parameters or multiparametric imaging [58] may warrant further investigation.

Several limitations of our study merit comment. Because of the retrospective nature of the study, a selection bias cannot be excluded. Our results related to the predictive value of SUV entropy and entropy need to be independently validated in longitudinal studies. Another caveat inherent in our study is the use of two methods (i.e., adaptive threshold and T45) for tumor delineation. Further studies are necessary to clarify the potential impact of the tumor delineation method on the prognostic significance of the indexes. Finally, we did not specifically investigate the impact of the reconstruction algorithms on the texture features [45] and their effect on survival.

## Conclusions

The results of our study indicate that texture features obtained with PET/HCT and PET/ACT showed limited differences and good levels of agreement regardless of the delineation method used. We also showed that texture parameters from PET/ACT are clinically useful in the prediction of survival in NSCLC patients and that SUV entropy and entropy are robust attenuation correction methods.

## Supporting Information

S1 Fig Results of Bland-Altman analysis. SUVmax (A), SUV mean (B), TLG (C), uniformity (D), dissimilarity (E), homogeneity (F), busyness (G), contrast (H), complexity (I), grey-level nonuniformity (J), zone-size nonuniformity (K), and high grey-level large zone emphasis (L).
(TIF)

S1 Table Results of Wilcoxon signed-ranks test for different PET parameters according to tumor location in the lung. (DOCX)

S2 Table Optimal cut-off values for different PET parameters in the prediction of 3-year disease-specific survival. (DOCX)

S3 Table Results of Wilcoxon signed-ranks tests for PET/HCT and PET/ACT parameters using T45 segmentation. (DOCX)

S4 Table Intraclass correlation coefficients and Bland-Altman analyses for PET parameters using T45 segmentation. (DOCX)

S5 Table Complete list of univariate and multivariate Cox regression analyses for the prediction of disease-specific survival using texture parameters. (DOCX)
