# Supplementary figures and images for: Respiration-Averaged CT for Attenuation Correction of PET Images – Impact on PET Texture Features in Non-Small Cell Lung Cancer Patients
Source: PLoS One. 2016 Mar 1;11(3):e0150509. doi: 10.1371/journal.pone.0150509 (PMC4773107; doi:10.1371/journal.pone.0150509)

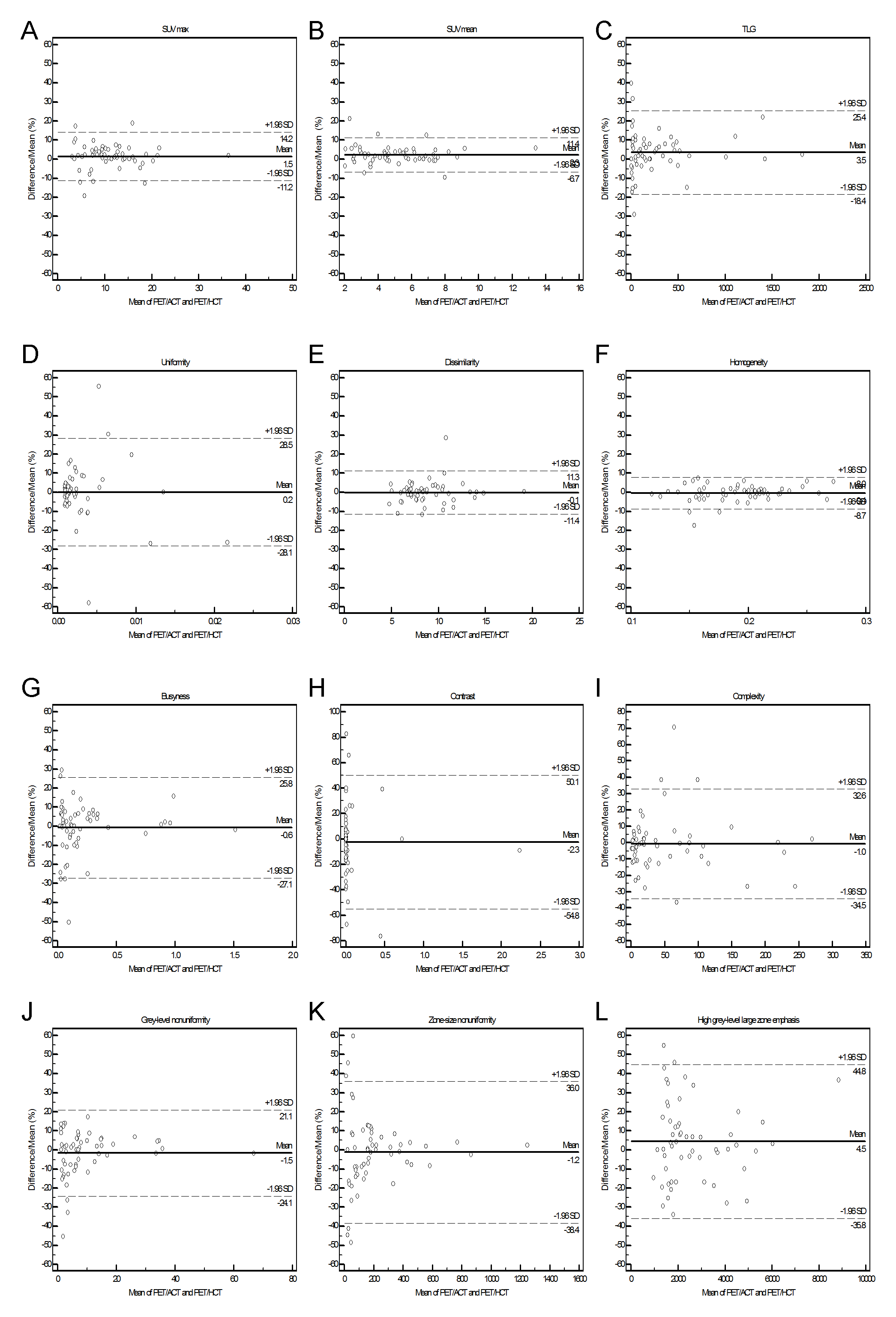

Supplement: S1 Fig — SUVmax (A), SUV mean (B), TLG (C), uniformity (D), dissimilarity (E), homogeneity (F), busyness (G), contrast (H), complexity (I), grey-level nonuniformity (J), zone-size nonuniformity (K), and high grey-level large zone emphasis (L). (TIF) [file pone.0150509.s001.tif]
