# Supplementary material for: Respiration-Averaged CT for Attenuation Correction of PET Images – Impact on PET Texture Features in Non-Small Cell Lung Cancer Patients
Source: PLoS One. 2016 Mar 1;11(3):e0150509. doi: 10.1371/journal.pone.0150509 (PMC4773107; doi:10.1371/journal.pone.0150509)
Supplement: S1 Table — (DOCX) [file pone.0150509.s002.docx]

**S1 Table. Results of Wilcoxon signed-ranks test for different PET parameters according to tumor location in the lung**

| **Variable** | **Lower lobes (n = 22)** | | | **Other lobes (n = 34)** | | |
| --- | --- | --- | --- | --- | --- | --- |
|  | **Median**  **(HCT vs. ACT)** | | ***P*** | **Median**  **(HCT vs. ACT)** | | ***P*** |
| **SUV_max_** | 10.19 | 10.72 | 0.005 | 10.17 | 10.22 | 0.313 |
| **SUV mean** | 4.88 | 4.87 | 0.009 | 4.56 | 4.68 | 0.001 |
| **TLG** | 144.4 | 156.7 | 0.013 | 99.0 | 98.7 | 0.015 |
| **Texture parameters** |  |  |  |  |  |  |
| **SUV entropy** | 3.80 | 3.78 | 0.733 | 3.75 | 3.76 | 0.304 |
| **Uniformity** | 0.002 | 0.002 | 0.733 | 0.002 | 0.002 | 0.597 |
| **Entropy** | 6.73 | 6.73 | 0.910 | 6.57 | 6.55 | 0.681 |
| **Dissimilarity** | 7.63 | 7.58 | 0.615 | 8.53 | 8.58 | 0.911 |
| **Homogeneity** | 0.203 | 0.203 | 0.808 | 0.183 | 0.180 | 0.955 |
| **Coarseness** | 0.020 | 0.019 | 0.548 | 0.024 | 0.026 | 0.970 |
| **Busyness** | 0.156 | 0.154 | 0.200 | 0.041 | 0.040 | 0.970 |
| **Contrast** | 0.0012 | 0.0014 | 0.140 | 0.002 | 0.002 | 0.940 |
| **Complexity** | 14.57 | 16.84 | 0.077 | 22.56 | 21.79 | 0.985 |
| **Grey-level nonuniformity** | 6.94 | 7.10 | 0.709 | 5.79 | 5.69 | 0.808 |
| **Zone-size nonuniformity** | 173.3 | 171.7 | 0.661 | 133.8 | 128.1 | 0.911 |
| **High grey-level large zone emphasis** | 2156 | 2469 | 0.322 | 1873 | 2110 | 0.239 |
| SUV: standardized uptake value; TLG: total lesion glycolysis. | | | | | | |
