# Supplementary material for: Respiration-Averaged CT for Attenuation Correction of PET Images – Impact on PET Texture Features in Non-Small Cell Lung Cancer Patients
Source: PLoS One. 2016 Mar 1;11(3):e0150509. doi: 10.1371/journal.pone.0150509 (PMC4773107; doi:10.1371/journal.pone.0150509)
Supplement: S2 Table — (DOCX) [file pone.0150509.s003.docx]

**S2 Table. Optimal cut-off values for different PET parameters in the prediction of 3-year disease-specific survival**

| **Variables** | **PET/HCT** | | | **PET/ACT** | | |
| --- | --- | --- | --- | --- | --- | --- |
|  | **Cut-off** | **AUC** | ***P*** | **Cut-off** | **AUC** | ***P*** |
| **SUV_max_** | > 7.367 | .588 | .288 | > 9.007 | .586 | .296 |
| **SUV mean** | > 4.426 | .586 | .289 | > 4.506 | .580 | .328 |
| **TLG** | > 113.96 | .682 | .017 | > 110.06 | .682 | .017 |
| **Texture parameters** |  |  |  |  |  |  |
| **SUV entropy** | > 3.842 | .726 | .001 | > 3.868 | .685 | .011 |
| **Uniformity** | > 0.0015 | .258 | .001 | > 0.0016 | .279 | .002 |
| **Entropy** | > 6.54 | .731 | .001 | > 6.51 | .721 | .002 |
| **Dissimilarity** | > 8.782 | .362 | .081 | > 9.631 | .357 | .067 |
| **Homogeneity** | > 0.178 | .635 | .082 | > 0.210 | .643 | .063 |
| **Coarseness** | > 0.023 | .298 | .006 | > 0.024 | .304 | .008 |
| **Busyness** | > 0.0721 | .667 | .027 | > 0.0753 | .672 | .021 |
| **Contrast** | > 0.0041 | .319 | .016 | > 0.0023 | .311 | .012 |
| **Complexity** | > 36.56 | .319 | .016 | > 37.00 | .324 | .019 |
| **Grey-level nonuniformity** | > 6.062 | .697 | .007 | >5.911 | .698 | .007 |
| **Zone-size nonuniformity** | > 156.43 | .718 | .002 | > 138.52 | .706 | .004 |
| **High grey-level large zone emphasis** | > 2076 | .618 | .127 | > 2004 | .632 | .090 |
| AUC: area under curve; SUV: standardized uptake value; TLG: total lesion glycolysis. | | | | | | |
