# Supplementary material for: Respiration-Averaged CT for Attenuation Correction of PET Images – Impact on PET Texture Features in Non-Small Cell Lung Cancer Patients
Source: PLoS One. 2016 Mar 1;11(3):e0150509. doi: 10.1371/journal.pone.0150509 (PMC4773107; doi:10.1371/journal.pone.0150509)
Supplement: S3 Table — (DOCX) [file pone.0150509.s004.docx]

**S3 Table. Results of Wilcoxon signed-ranks tests for PET/HCT and PET/ACT parameters using T45 segmentation**

| **Variables** | **PET/HCT** | | | **PET/ACT** | | | ***P*** |
| --- | --- | --- | --- | --- | --- | --- | --- |
|  | **Mean** | **SD** | **Range** | **Mean** | **SD** | **Range** |  |
| **SUV_max_** | 11.12 | 5.95 | 3.03-36.05 | 11.29 | 6.03 | 3.08-36.74 | 0.009 |
| **SUV mean** | 6.60 | 3.44 | 1.67-19.44 | 6.71 | 3.49 | 1.70-19.87 | 0.003 |
| **TLG** | 163.1 | 211.7 | 1.5-883.6 | 170.9 | 224.9 | 1.7-1046.9 | <0.001 |
| **Texture parameters** |  |  |  |  |  |  |  |
| **SUV entropy** | 3.71 | 0.26 | 2.66-4.02 | 3.72 | 0.26 | 2.74-4.01 | 0.478 |
| **Uniformity** | 0.004 | 0.008 | 0.001-0.06 | 0.004 | 0.005 | 0.001-0.036 | 0.316 |
| **Entropy** | 6.05 | 0.78 | 2.86-7.14 | 6.08 | 0.75 | 3.33-7.14 | 0.233 |
| **Dissimilarity** | 10.63 | 3.43 | 5.64-22.80 | 10.56 | 3.25 | 5.77-21.86 | 0.269 |
| **Homogeneity** | 0.17 | 0.03 | 0.11-0.24 | 0.17 | 0.03 | 0.10-0.24 | 0.740 |
| **Coarseness** | 0.031 | 0.018 | 0.004-0.073 | 0.030 | 0.02 | 0.004-0.068 | 0.210 |
| **Busyness** | 0.173 | 0.216 | 0.020-1.070 | 0.167 | 0.189 | 0.020-1.081 | 0.284 |
| **Contrast** | 0.871 | 6.132 | 0.000-45.92 | 0.377 | 2.378 | 0.000-17.76 | 0.320 |
| **Complexity** | 75.92 | 82.34 | 2.76-421.4 | 72.89 | 75.00 | 2.50-377.8 | 0.080 |
| **Grey-level nonuniformity** | 5.99 | 6.78 | 1.00-36.86 | 5.96 | 6.56 | 1.00-37.32 | 0.567 |
| **Zone-size nonuniformity** | 135.2 | 142.9 | 5.3-766.7 | 135.8 | 138.8 | 10.0-721.6 | 0.894 |
| **High grey-level large zone emphasis** | 2273 | 1609 | 497-6000 | 2266 | 1610 | 467-6503 | 0.766 |
| SD: standard deviation; SUV: standardized uptake value; TLG : total lesion glycolysis | | | | | | | |
