# Supplementary material for: Respiration-Averaged CT for Attenuation Correction of PET Images – Impact on PET Texture Features in Non-Small Cell Lung Cancer Patients
Source: PLoS One. 2016 Mar 1;11(3):e0150509. doi: 10.1371/journal.pone.0150509 (PMC4773107; doi:10.1371/journal.pone.0150509)
Supplement: S4 Table — (DOCX) [file pone.0150509.s005.docx]

**S4 Table. Intraclass correlation coefficients and Bland-Altman analyses for PET parameters using T45 segmentation**

| **Variables** | **Intraclass Correlation Coefficient (ICC)** | | | **Bland-Altman analysis** | | |
| --- | --- | --- | --- | --- | --- | --- |
|  | **ICC** | **95% CI** | **Precision (%)** | **Mean** | **Variation (%)** | **LRL^a^–URL^b^ (%)** |
| **SUV_max_** | 0.993 | 0.989-0.996 | 0.35 | 1.5 | 25.4 | -11.2-14.2 |
| **SUV mean** | 0.994 | 0.989-0.996 | 0.35 | 1.8 | 23.5 | -9.9-13.6 |
| **TLG** | 0.992 | 0.986-0.995 | 0.45 | 4.7 | 33.6 | -12.1-21.5 |
| **Texture parameters** |  |  |  |  |  |  |
| **SUV entropy** | 0.958 | 0.930-0.975 | 2.25 | 0.3 | 11.5 | -5.5-6.0 |
| **Uniformity** | 0.878 | 0.800-0.926 | 6.30 | -2.5 | 49.3 | -27.1-22.2 |
| **Entropy** | 0.988 | 0.980-0.993 | 0.65 | 0.6 | 10.4 | -4.60-5.80 |
| **Dissimilarity** | 0.981 | 0.967-0.989 | 1.10 | -0.4 | 24.4 | -12.6-11.8 |
| **Homogeneity** | 0.973 | 0.955-0.984 | 1.45 | 0.1 | 19.5 | -9.6-9.9 |
| **Coarseness** | 0.950 | 0.916-0.970 | 2.70 | -3.2 | 55.0 | -30.7-24.3 |
| **Busyness** | 0.930 | 0.884-0.959 | 3.75 | 2.1 | 70.1 | -32.9-37.2 |
| **Contrast** | 0.672 | 0.499-0.794 | 14.75 | -3.9 | 91.7 | -49.7-42.0 |
| **Complexity** | 0.983 | 0.971-0.990 | 0.95 | -1.6 | 68.5 | -35.9-32.6 |
| **Grey-level nonuniformity** | 0.989 | 0.982-0.994 | 0.60 | 0 | 39.9 | -20.0-19.9 |
| **Zone-size nonuniformity** | 0.993 | 0.988-0.996 | 0.40 | 1.2 | 77.2 | -37.4 - 39.8 |
| **High grey-level large zone emphasis** | 0.937 | 0.896-0.963 | 3.35 | 0.6 | 101.8 | -50.3-51.5 |
| SUV: standardized uptake value; TLG: total lesion glycolysis; ^a^LRL: lower reproducibility limit; ^b^URL: upper reproducibility limit. | | | | | | |
