# Supplementary material for: Respiration-Averaged CT for Attenuation Correction of PET Images – Impact on PET Texture Features in Non-Small Cell Lung Cancer Patients
Source: PLoS One. 2016 Mar 1;11(3):e0150509. doi: 10.1371/journal.pone.0150509 (PMC4773107; doi:10.1371/journal.pone.0150509)
Supplement: S5 Table — (DOCX) [file pone.0150509.s006.docx]

**S5 Table. Complete list of univariate and multivariate Cox regression analyses for the prediction of disease-specific survival using texture parameters**

| **Texture parameters** | **PET/HCT** | | | **PET/ACT** | | |
| --- | --- | --- | --- | --- | --- | --- |
|  | **HR** | **95% CI** | ***P*** | **HR** | **95% CI** | ***P*** |
| **Univariate Cox** |  |  |  |  |  |  |
| **SUV entropy** | 2.69 | 1.37-5.29 | 0.004 | 2.49 | 1.24-4.99 | 0.010 |
| **Uniformity** | 0.40 | 0.20-0.79 | 0.008 | 0.38 | 0.19-0.76 | 0.007 |
| **Entropy** | 2.81 | 1.34-5.92 | 0.006 | 2.81 | 1.34-5.92 | 0.006 |
| **Coarseness** | 0.38 | 0.19-0.76 | 0.007 | 0.38 | 0.19-0.76 | 0.007 |
| **Contrast** | 2.22 | 1.06-4.65 | 0.035 | 2.34 | 1.14-4.80 | 0.021 |
| **Grey-level nonuniformity** | 2.10 | 1.04-4.24 | 0.039 | 2.36 | 1.17-4.78 | 0.017 |
| **Zone-size nonuniformity** | 2.43 | 1.21-4.86 | 0.012 | 2.66 | 1.31-5.41 | 0.007 |
| **Multivariate Cox** |  |  |  |  |  |  |
| **SUV entropy** | 2.69 | 1.32-5.45 | 0.006 | 2.77 | 1.32-5.83 | 0.007 |
| **Uniformity** | 0.44 | 0.21-0.93 | 0.033 | 0.40 | 0.19-0.85 | 0.017 |
| **Entropy** | 2.69 | 1.23-5.89 | 0.013 | 2.69 | 1.23-5.89 | 0.013 |
| **Coarseness** | 0.34 | 0.15-0.79 | 0.012 | 0.34 | 0.15-0.79 | 0.012 |
| **Contrast** | 2.39 | 1.08-5.31 | 0.032 | 2.34 | 1.06-5.14 | 0.035 |
| **Grey-level nonuniformity** | 2.07 | 0.96-4.48 | 0.065 | 2.69 | 1.17-6.17 | 0.020 |
| **Zone-size nonuniformity** | 2.11 | 1.01-4.41 | 0.046 | 2.18 | 1.03-4.64 | 0.043 |

HR: hazard ratio; CI: confidence interval; SUV: standardized uptake value.
